# Antiplatelet agents for prevention of pre-eclampsia and its consequences: a systematic review and individual patient data meta-analysis

**DOI:** 10.1186/1471-2393-5-7

**Published:** 2005-03-18

**Authors:** The Perinatal Antiplatelet Review of International Studies (PARIS) Collaboration Steering Group on behalf of the PARIS Collaboration, The PARIS Collaboration

**Affiliations:** 1 Centre for Perinatal Health Services Research, Building DO2, University of Sydney, NSW, 2006, Australia

## Abstract

**Background:**

There is now good evidence that antiplatelet agents (principally low dose aspirin) prevent pre-eclampsia, a leading cause of morbidity and mortality for pregnant women and their babies. A Cochrane Review identified moderate, but clinically important, reductions in the relative risks of pre-eclampsia (19%), preterm birth (7%) and perinatal mortality (16%) in women allocated antiplatelets, rather than placebo or no antiplatelet.

Uncertainty remains, however, about whether some women (in terms of risk) benefit more than others, what dose of aspirin is best and when in pregnancy treatment should ideally start. Rather than undertake new trials, the best way to answer these questions is to utilise existing individual patient data from women enrolled in each trial.

**Methods/Design:**

Systematic review with meta-analysis based on individual patient data. This involves the central collection, validation and re-analysis of thoroughly checked data from individual women in all the available randomised trials.

The objective is to confirm that antiplatelet agents, given during pregnancy, will reduce the incidence of pre-eclampsia. The review will then determine the size of this effect, and whether antiplatelets delay the onset of pre-eclampsia or its impact on important outcomes for women and their babies. It will also explore whether the effect of antiplatelets differs by womens' risk profile; when commenced during pregnancy; and/or by dose.

**Discussion:**

The PARIS (Perinatal Antiplatelet Review of International Studies) Collaboration has been formed to undertake the review. This will be the first individual patient data review in the perinatal field. Final results should be available by 2006–7.

## Background

### Clinical significance of pre-eclampsia

World-wide, over half a million women die each year of pregnancy related causes with 99% of these occurring in low resource countries [1,2]. An estimated 10–15% of these maternal deaths are associated with hypertensive disorders of pregnancy [3].

High blood pressure is common during pregnancy: approximately one in ten women will have their blood pressure recorded as above normal at some point before delivery [4]. For women who develop raised blood pressure but have no other complications, pregnancy outcome is similar to that for women with normal blood pressure. Pre-eclampsia, a multi-system disorder of pregnancy usually associated with high blood pressure (hypertension) and proteinuria, complicates 2–8% of pregnancies [5]. It can affect the mother's organs, leading to problems in liver, kidneys and brain, and to abnormalities of the clotting system. As the placenta is also involved, there are increased risks for the baby. The most common problems are poor fetal growth due to inadequate blood supply through the damaged placenta, and prematurity, related either to the spontaneous onset of pre-term labour or the need for an early, elective delivery.

Although the outcome for most women is good, pre-eclampsia and eclampsia (the rare occurrence of seizures superimposed on the syndrome of pre-eclampsia) are major causes of maternal mortality. In low resource countries pre-eclampsia and eclampsia account for 10–15% of maternal deaths [3] whilst in high resource countries pre-eclampsia is consistently a leading cause of maternal mortality [6,7]. Perinatal mortality is also increased [8,9]. There is little good quality information about morbidity for either mother or baby, but it is likely that this too is high. For example, pre-eclampsia accounts for about one fifth of antenatal admissions [10], two thirds of referrals to day care assessment units [11] and a quarter of obstetric admissions to intensive care units [12]. Pre-eclampsia is an antecedent for up to 19% of pre-term births [13] and 12% of growth restricted babies [14]. Also of note is the high rate of intrauterine growth restriction (20–25%) in pregnancies complicated by pre-eclampsia and the possible lifelong health effects due to prenatal programming [15].

Pre-eclampsia is a multi-factorial condition. Although its aetiology remains unclear, there have been significant advances in the understanding of the pathophysiology of the disorder. The primary lesion is thought to be deficient trophoblast invasion of the maternal spiral arteries in the second trimester, leading to underperfusion of the uteroplacental circulation and placental ischaemia [16]. The resulting placental damage is thought to lead to release of factors into the maternal circulation, which are responsible for the maternal syndrome. Activation of platelets and the clotting system may occur early in the course of the disease, before clinical symptoms develop [17,18]. Deficient intravascular production of prostacyclin, a vasodilator, with excessive production of thromboxane, a platelet-derived vasoconstrictor and stimulant of platelet aggregation [19,20], have also been demonstrated to occur in pre-eclampsia. These observations have led to the hypothesis that antiplatelet agents, low dose aspirin (<300 mg/day) in particular, might prevent or delay the development of pre-eclampsia or reduce its severity and the risk of adverse events.

It is further hypothesised that the effect of antiplatelets may be different if treatment is started before placental implantation is complete. [21]. If this hypothesis were correct, the greatest benefit should be seen in women who started treatment before 16 weeks gestation, with the effect attenuating with later onset of treatment. Similarly, it remains unclear as to the most appropriate dose of antiplatelet therapy for the prevention of pre-eclampsia in order to maximise benefits whilst minimising harms [22]. It has been suggested that low doses of aspirin may selectively inhibit the cyclo-oxygenase pathway in platelet production but not in vessel wall endothelium thereby diminishing the synthesis of thromboxane but not of prostacycline. A higher dose may inhibit both thromboxane and prostacycline thereby neutralising the effect of the intervention [20]. However, there is also limited evidence from randomised trials that a higher dose of aspirin may effect a greater reduction in the risk of pre-eclampsia [23].

Although it is known that pre-eclampsia is a multi-system disorder, the relationship between the placental pathology and maternal endothelial response is not fully understood. Numerous maternal factors can predispose to the disorder, such as previous pre-eclampsia, diabetes, renal disease, chronic hypertension or other risk factors [24]. The syndrome known as pre-eclampsia may also be more than one disease, each with distinct origins, pathologic characteristics and natural history, rather than one fundamental process with varying degrees of clinical severity [25,26]. Hence, the ability to assess the affect of antiplatelets on women with individual risk factors, or a series of risk factors, is of great relevance to clinicians and women. A meta-analysis based on data from individual women will enable the exploration of these hypotheses.

### Randomised trials of antiplatelet agents

The effects of antiplatelet agents were first evaluated in small randomised trials, which reported striking reductions in the risk of hypertension and proteinuria [27-29]. These trials were too small to provide reliable information about other more substantive outcomes, such as perinatal mortality and preterm birth. Also, there was no information about the potential hazards of antiplatelet therapy, such as a possible increased risk of bleeding for both the woman and her baby, or possible effects on infant and child development. The promising results of these early trials led to several large studies around the world. Before these could be completed, however, the use of low dose aspirin had already become relatively widespread for women considered at increased risk of pre-eclampsia. Results of the larger trials were disappointing, as they failed to confirm any statistically significant reductions in substantive outcomes [30]. Nevertheless, the first Cochrane Review of these trials demonstrated that, when taken together, there are modest, but clinically important benefits [31,32].

### Summary of systematic review of aggregate data in 2004

The updated Cochrane Review identified 51 trials with over 36,500 pregnant women evaluating antiplatelet agents, principally low dose aspirin, for the prevention of pre-eclampsia [23]. Nine of these trials included over 1000 women, and 15 involved less than 50 women. Fifty-one studies were excluded, mostly due to the non-availability of clinically relevant data. Aggregate data from the included trials demonstrated a 19% reduction (RR 0.81, 95% CI 0.75–0.88) in the risk of developing pre-eclampsia associated with the use of antiplatelet agents, rather than placebo or no antiplatelet. There was also a small (7%) reduction in the risk of pre-term birth (RR 0.93, 95% CI 0.89–0.98) and a 16% reduction in the risk of the baby dying (RR 0.84 95% CI 0.74–0.96). Based on the average risk of women included in these trials, about 70 women would have to be treated to prevent one case of pre-eclampsia, and 240 to prevent one baby death.

These effects are much smaller than had initially been hoped for but, nevertheless, potentially they have considerable public health importance. The conclusion from this aggregate data review was that for most low-risk women large numbers of women would need to be treated with antiplatelets agents to prevent one episode of either pre-eclampsia or perinatal death. Whether there are specific high risk sub-groups of pregnant women for whom there might be greater benefits, remains unclear, as does the best time to initiate treatment, and at what dose. **The aim is now to extend this review based on aggregate data, to utilise the available data for every individual woman in each trial to help address these remaining questions. **The use of individual data for each woman will allow for more powerful and flexible analysis of both subgroups and outcomes [33,34]. This review will therefore provide more specific information to guide the care of women at risk of pre-eclampsia.

### Limitations of the review using published, aggregate data

• Many studies were excluded because publications did not report sufficient information to allow them to be included in the review.

• The published aggregate data is variable in the completeness of outcome reporting and in definitions used between trials.

• The aggregate data meta-analyses were restricted to analysing outcomes for complete trials. As many trials included women with a wide range of risk profiles at trial entry, trials could only be defined by average values. Such aggregated outcomes such as 'proportion of women developing pre-eclampsia' conceal a range of severity of disorder and so it was not possible to explore fully whether the effectiveness of antiplatelet therapy differed according to risk.

• It was also difficult to make precise recommendations about when to start treatment. While most trials could generally be classified as starting treatment at earlier or later in gestational ages, there was a wide within-study variation that could not be explored with aggregate data analyses.

• There is a markedly skewed distribution of effect estimates for each trial around the summary effect on pre-eclampsia, with more small positive trials than small negative trials. This suggests the possibilities of publication bias or that the differences in the characteristics of the women enrolled in small and large studies has an important influence on the effects of antiplatelet agents.

### Ways of overcoming these limitations by using individual patient data

• Obtaining individual patient data from previously excluded trials will allow assessment of whether these trials are eligible for inclusion, both in terms of available outcomes and methodological quality. This will potentially increase the power and scope of the analyses.

• Trial level information obtained by direct discussion with the trialists enables clarification of the definitions and measurements used. Data for measured, but previously unreported, outcomes can also often be obtained and trialists, who are part of a Collaborative group, may be able to provide missing outcome data. Furthermore, we will be able to apply common definitions across trials based on each woman's baseline and clinical data. For example, where possible, we will define pre-eclampsia based on a set of uniform criteria.

• Subgroup analyses will be performed for a number of different risk factors using individual patient's specific risk data, rather than using the aggregated risk profile of all women enrolled in a particular trial.

• By obtaining gestational age at randomisation for each individual woman, more precise patient-based analyses can be performed exploring whether gestation at treatment commencement alters the effectiveness of antiplatelets.

• Formation of a Collaborative group, is likely to lead to a more complete identification of all relevant trials, including those previously unpublished. This may help overcome the potential for publication bias. The patient-level data will also allow us to explore whether there were important differences in the characteristics of women enrolled in small and large trials.

## Methods/design

### Objectives

The objective of this review is to confirm that antiplatelet agents, given during pregnancy, reduce the incidence of pre-eclampsia. The review will then determine the size of this effect, and whether antiplatelets reduce the severity of pre-eclampsia and/or its impact on important outcomes for women and their babies. It will also explore whether the effect of antiplatelets differs by womens' risk profile, when commenced during pregnancy, and/or by dose.

The main questions to be addressed in this review are:

• Do antiplatelet agents, primarily low dose aspirin, have clinically important benefits for women at risk of developing pre-eclampsia and their babies?

Investigation of this hypothesis will also explore whether the treatment effect differs, in a clinically meaningful way, between women with different risk factors such as those with a history of early onset pre-eclampsia, renal disease, diabetes, chronic hypertension, or autoimmune disease.

• Does the planned dose of aspirin affect outcome in terms of preventing or delaying the onset of pre-eclampsia or other adverse outcomes, such as preterm birth or perinatal death?

• Do the effects of aspirin differ according to gestation at onset of treatment?

### Identifying studies

The search strategy to identify potentially eligible studies will include a search of the register of trials developed and maintained by the Cochrane Collaboration Pregnancy and Childbirth Review Group. Details of how this register is maintained are available elsewhere [35,36], but it involves extensive searching of bibliographic databases such as MEDLINE, *The Cochrane Controlled Trials Register *and hand searching of relevant journals. Trialists will be asked if they know of any further studies. [See Additional file 1] for the list of trials potentially eligible for inclusion. In addition, all members of the Collaborative Group will be asked to notify any unpublished trials of which they are aware.

### Inclusion and exclusion criteria for studies

The inclusion and exclusion criteria for the types of study designs, participants, interventions and data completeness to be included in the review are listed below. Each potentially eligible study will be assessed independently by two members of the Secretariat, unblinded to the trial's identity. Any differences of opinion regarding the assessment of the inclusion criteria will be resolved by discussion between the two assessors. If differences cannot be resolved, a third member of the Secretariat will be asked to assess the study. If individual patient data are unavailable from an eligible trial, the trial will remain included in the review and aggregate data will be used.

#### a. Study design

Studies will be included in the review if they were randomised trials. Quasi-random study designs, such as those using alternate allocation, will be excluded. The level of allocation concealment within each trial will be assessed according to the criteria outlined in the Cochrane Handbook [37], and classified as either adequate, unclear or clearly inadequate. These assessments will be made together with the outcomes of thorough data checking procedures.

#### b. Participants

Participants will be pregnant women at risk of developing pre-eclampsia. Women who started treatment postpartum will be excluded, as will those who already have a diagnosis of pre-eclampsia at trial entry (defined as hypertension with new onset proteinuria after 20 weeks gestation, not due to renal disease).

#### c. Interventions

The interventions will be any comparisons of an antiplatelet agent (such as low dose aspirin or dipyridamole), or any combination of antiplatelet agents, compared with placebo or no antiplatelet agent. This is regardless of dose, mode of administration and irrespective of whether the antiplatelet is in combination with another drug. Trials that assessed only physiological outcomes following a short duration of intended therapy will be excluded.

#### d. Completeness of follow-up

The main analyses will include all trials that fulfill the previous inclusion criteria, regardless of completeness of follow-up. Sensitivity analyses will be undertaken to assess the effect of the inclusion of data from trials where only small numbers of enrolled participants have available outcome data. The threshold for an acceptable level of data completeness may vary by outcome. For example, for long-term follow-up of women and children, data may be included if follow-up was less than 80% provided that substantive bias between the groups was unlikely. Other outcomes from each trial may only be included in the analysis if available for 80% or more of women.

### Data collection, data management and confidentiality

The individual patient data provided by the Collaborators will be de-identified, re-coded as required and stored in a custom-designed Microsoft ACCESS database. It will not include any patient identifying information such as names or addresses. Electronic data will be located on a secure, password protected network server. Copies of hardcopy data will be stored in locked filing cabinets until converted into electronic format, and will then be securely destroyed. Only authorised personnel will have access to the data. All data will be securely stored and archived according to the policies of the major funder, the Australian National Health and Medical Research Council.

The data will be checked with respect to range, internal consistency, consistency with published reports and missing items. Trial details such as randomisation methods, and dose and timing of the interventions will be cross-checked against any published reports, trial protocols and data collection sheets. Integrity of the randomisation process will be examined by reviewing the chronological randomisation sequence and pattern of assignment, as well as the balance of prognostic factors across treatment groups (taking into account stratification factors). Inconsistencies or missing data will be discussed with the individual trialists and attempts will be made to resolve any problems by consensus. Each trial will be analysed individually, and the resulting analyses and trial data will be sent to the trialists for verification.

### Data items requested from the trialists

There has been extensive consultation with the PARIS Collaborative Group regarding what data items to collect for each woman in the analyses. The following section contains the list of data items requested from trialists, which has been compiled following this consultation. More detailed definitions for the data items listed below can be found in Tables 1 and 2. Details of the suggested coding for each of the following variables can be found in [see Additional file 2]. A formal request for the provision of the individual patient data was sent in April 2004 [see Additional file 3].

**Table 1:** Key definitions for enrolment characteristics

**Variable**	**Definition**
gestational hypertension	*de novo *systolic BP ≥ 140 mmHg *and/or *diastolic BP ≥ 90 mmHg after 20 weeks' gestation, without proteinuria
severe hypertension	systolic BP ≥ 160 mmHg *and/or *diastolic BP ≥ 110 mmHg
proteinuria	≥ 1+ on dipstick, *or *≥ 300 mg/24 hours, *or *spot urine protein/creatinine ratio ≥ 30 mg/mmol
pre-eclampsia (for women normotensive at trial entry)	*de novo *systolic BP ≥ 140 mmHg *and/or *diastolic BP ≥ 90 mmHg after 20 weeks' gestation with new-onset proteinuria as described above
pre-eclampsia (for women with chronic hypertension at trial entry)	new-onset proteinuria as described above
pre-eclampsia (for women with chronic hypertension and proteinuria at trial entry)	signs and symptoms of superimposed pre-eclampsia after 20 weeks' gestation, for example worsening of hypertension or proteinuria
early onset proteinuria	proteinuria as defined above, occurring ≤ 33 weeks + 6 days of gestation
early onset pre-eclampsia	hypertension and early onset proteinuria as described above
chronic hypertension	*Essential hypertension*: BP ≥ 140/90 mmHg pre-conception or in first half of pregnancy without an underlying cause, *or Secondary hypertension*: hypertension associated with renal, renovascular, cardiac and endocrine disorders
intrauterine growth restriction (IUGR) *or *small for gestational age (SGA)	growth below the 3^rd ^centile, *or *as defined in the individual trial
miscarriage / fetal death	any death in utero
perinatal death	death in utero or within the first 7 days of life
neonatal death	live born and any reported death within the first 28 days

**Table 2:** Key definitions for outcome measures

**Main outcomes**	**Definition**
pre-eclampsia	as defined in Table 1
pregnancy loss / neonatal death	miscarriage, fetal death or death of a liveborn infant before hospital discharge
pre-term birth	*pre-term birth*: ≤ 37 weeks + 6 days of gestation *moderately pre-term birth*: ≤ 33 weeks + 6 days of gestation *extremely pre-term birth*: ≤ 27 weeks + 6 days of gestation
small for gestational age (SGA) infant	infant with birth-weight below the 3^rd ^centile, *or *as defined in the individual trial
pregnancy with serious adverse outcome	pregnancy with any of the above main outcomes for the woman or any fetus/baby, or the death of the woman. If sufficient data available, severe maternal morbidity will also be included in this definition.
**Other outcomes**	**Definition**
early onset pre-eclampsia	as defined in Table 1
maternal death	death during pregnancy or up to 42 days after termination of the pregnancy
antepartum haemorrhage	any vaginal bleeding before the onset of labour
placental abruption	clear evidence of placental separation
severe maternal morbidity	including eclampsia, HELLP syndrome, DIC, pulmonary oedema, liver failure, renal failure or CVA/stroke
infant death	live born and any reported death from 29 days to 1 year of life or after hospital discharge
neonatal bleeding	abnormal bleeding in the neonatal period including periventricular haemorrhage, gastrointestinal, umbilical or other sites

#### a. Characteristics of trials

1 informed consent

2 dates trial opened and closed to accrual

3 total number of women randomised

4 treatments used in each arm of the trial

5 intended duration of treatments

6 definitions of key outcomes used in the trial

7 method of random allocation

8 stratification factors used

9 methods of allocation concealment

#### b. Characteristics of enrolled women at trial entry

1 unique identifier for the enrolled woman, coded for anonymity

2 date of randomisation

3 gestational age at randomisation, or best estimate of expected date of delivery or last menstrual period

4 woman's date of birth or age

5 any previous pregnancy

6 blood pressure (systolic, diastolic, whether diagnosed as raised)

7 presence of proteinuria

8 presence of oedema

9 risk factors – multiple pregnancy, autoimmune disease, renal disease, diabetes, chronic hypertension, previous gestational hypertension or pre-eclampsia/eclampsia (including early onset disease), family history of pregnancy-related hypertensive disorders, previous fetal growth restriction, previous perinatal death, abnormal uterine artery Doppler flow and abnormalities on other diagnostic tests

#### c. Maternal data items

1 hypertension during pregnancy (highest blood pressure, diagnosis of severe hypertension)

2 proteinuria during pregnancy (including date and/or gestation at onset)

3 oedema during pregnancy

4 pre-eclampsia

5 drug treatment for pre-eclampsia or hypertension

6 severe maternal morbidity (including eclampsia, renal failure, disseminated intravascular coagulation, liver failure, HELLP syndrome, stroke)

7 onset of labour (spontaneous or induced or pre-labour caesarean section)

8 mode of delivery (vaginal, vaginal assisted, caesarean section)

9 antepartum haemorrhage (all, placental abruption)

10 postpartum haemorrhage and/or estimated blood loss at delivery

11 maternal mortality

#### d. Fetal / neonatal / child data items (for each fetus)

1 gestational age at birth and/or date of birth

2 birthweight

3 gender

4 small for gestation age (as defined within each trial: including centile charts and cut-off point used)

5 miscarriage or stillbirth: date and/or gestational age at death/loss

6 neonatal or infant death: date and/or age at death

7 admission to special care baby unit or neonatal intensive care unit

8 use of assisted ventilation

9 number of days in hospital or date of hospital discharge

10 neonatal bleeding (for example, periventricular haemorrhage)

11 child growth and development (such as cerebral palsy, blindness, deafness, significant cognitive delay – as defined within each trial)

### Planned analyses

This section contains a summary of the planned analyses. The full, detailed analysis plan will be discussed and agreed upon by the Collaborators before any data have been analysed.

Analysis will aim to be of all women ever randomised and will be based on intention to treat. In the main analyses a two stage approach will be taken. Outcomes will be analysed in their original trial and then these separate results will be combined to give an overall measure of effect. A fixed effect model will be used and the assumption of homogeneity of treatment effects will be tested using the chi squared test. The I^2 ^statistic will also be used to assess consistency of results.

#### 1. Outcomes to be analysed

The main analyses comparing the effect of antiplatelet agents with placebo or no antiplatelet agents will be undertaken for all outcomes listed below, for the woman and any fetus/baby. The planned sub-group and sensitivity analyses will be restricted to the designated main outcomes listed as follows:

##### a. Main outcomes

• pre-eclampsia

• pregnancy loss / neonatal death

• pre-term birth

• small for gestational age infant

• pregnancy with serious adverse outcome

##### b. Other outcomes

• early onset pre-eclampsia

• maternal death

• severe maternal morbidity

• antepartum haemorrhage

• placental abruption

• induction of labour

• Caesarean section delivery

• postpartum haemorrhage

• gestation at delivery

• infant admission to special care or neonatal intensive care unit

• infant required assisted ventilation

• neonatal bleeding

• infant death

#### 2. Planned sub-group analyses

The planned sub-group analyses will be restricted to the designated main outcomes unless there are clear indications for expanding the analyses further.

##### a. Trial-level characteristics

The effect of antiplatelet therapy may vary across the trials in the meta-analysis because they have used different agents in different ways. To explore this further, analyses are planned whereby trials will be grouped according to the agent used and by dose. These analyses will focus on the main outcomes. Trials will be classified into subsets based on the following:

###### (i) type of antiplatelet(s)

Trials will be grouped by the type of antiplatelet agent (trials that used aspirin alone, trials that used other antiplatelet agents, trials that used both aspirin and other antiplatelets agents) given as the active treatment.

###### (ii) daily dose of aspirin

In trials that used aspirin alone, trials will be grouped by planned aspirin dose (<75 mg, 75–149 mg, ≥ 150 mg).

##### b. Patient-level characteristics

One of the strengths of individual patient data reviews is that it allows us to assess eligibility and outcome using individual women's characteristics. Subgroup analyses will explore whether any particular risk factors act as effect modifiers. That is, are there any particular types of women who benefit more or less from antiplatelet agents? These analyses will take account each individual woman's own characteristics, rather than relying on summary measures of the 'average' risk profile of all participants in an individual trial.

Analyses will be undertaken to explore whether there are any particular types of women who benefit more or less from antiplatelet agents based on the following criteria:

###### (i) risk factor profile for pre-eclampsia at trial entry

Women normotensive at trial entry:

- **previous hypertensive disorders of pregnancy**: previous early onset (≤33 weeks + 6 days gestation) pre-eclampsia or eclampsia / previous pre-eclampsia / previous gestational hypertension / no previous hypertensive disorders of pregnancy / no previous pregnancy but family history of hypertensive disorders of pregnancy / no previous pregnancy and no family history of hypertensive disorders of pregnancy

- **diabetes**: pre-existing diabetes at enrolment / no pre-existing diabetes at enrolment

- **renal disease**: pre-existing renal disease / no pre-existing renal disease

- **autoimmune disease**: autoimmune disease / no autoimmune disease

- **multiple pregnancy**: multiple pregnancy / singleton pregnancy

- **maternal age**: <20 years / 20–35 years / >35 years. Maternal age may also be analysed as a continuous variable.

- **diagnostic test results**: abnormal uterine artery Doppler scan / other diagnostic test abnormalities / no abnormal diagnostic test results

- **previous SGA**: previous small for gestational age infant / previous infant not small for gestational age / no previous infant

- **primigravida**: first pregnancy with no other risk factors / first pregnancy with one or more risk factors / second or subsequent pregnancy with one or more risk factors / second or subsequent pregnancy with no risk factors

Women with hypertension at trial entry:

- **hypertension: **gestational hypertension / chronic hypertension

###### (ii) gestation at trial entry

To determine whether antiplatelet agents are differentially effective if given earlier in pregnancy and to determine the magnitude of any difference, gestational age at randomisation will be primarily analysed as a continuous variable in regression analyses. However, a subgroup analysis with women classified according to the following categories may also be performed: <16 weeks, 16–19 completed weeks, 20–23 completed weeks, 24–27 completed weeks, ≥28 weeks gestation. If numbers are insufficient for any category, categories will be combined.

#### 3. Planned sensitivity analyses

a. To assess whether results are robust to the inclusion or exclusion of particular types of trials or patients, the following sensitivity analyses will be conducted:

• exclusion of trials that did not use a placebo for the control group

• exclusion of trials of small size

• exclusion of poor quality trials (assessed by adequacy of allocation concealment, blinding, completeness of follow-up and other data checking procedures)

b. To assess whether results are robust to different methods of analysis or different definitions of pre-eclampsia [38,39], the following sensitivity analyses will be conducted:

• comparison of analyses using random effects and fixed effect models

• comparison of analyses using individual patient data (IPD) only with analyses using individual patient data and aggregate data where IPD unavailable

• comparison of analyses using different definitions of pre-eclampsia

These sensitivity analyses will be carried out for the main outcomes.

#### 4. Additional analyses

Depending on what data are available, the level of heterogeneity encountered and available time a one-stage modeling approach may also be undertaken to further explore important key outcomes as appropriate.

### Ethical considerations

Participants in the individual trials have previously given informed consent to participate in their respective trial. The data for this project are to be used for the purpose for which they were originally collected and are available through an agreement between all trialists of the PARIS Collaboration. These trialists remain the custodians of their original individual trial data at all times. Data are provided on the stipulation that all trials have received ethical clearances from their relevant bodies.

### Project management

Membership of the PARIS Collaboration will be representative(s) from each of the trials contributing data to the review with an accompanying project coordination and data management structure as described in this section.

The membership and responsibilities of each of these management groups is as follows:

#### a. Steering Group

The Steering Groupwill be responsible for project management decisions and will meet approximately 4–6 times per year, usually via teleconference. Membership: D Henderson-Smart^1 ^(co-chair), L Duley^2 ^(co-chair), L Askie^1 ^(project coordinator), M Showell^1 ^(project administrator), B Farrell^2^, L Stewart^3^, M Clarke^4^, J King^5^, C Roberts.^1 ^The first six members act as the **Secretariat**.

^1 ^Centre for Perinatal Health Services Research, University of Sydney, Australia;

^2 ^Resource Centre for Randomised Trials, University of Oxford, Oxford, UK;

^3 ^Medical Research Council Clinical Trials Unit, London, UK;

^4 ^UK Cochrane Centre, Oxford, UK;

^5 ^Royal Women's Hospital, Melbourne, Australia.

#### b. Advisory Group

The aim of the Advisory Group is to facilitate representative input from the Collaborative Group to the Steering Group. Membership of the Advisory Group will include people who have contributed to the trials included in the project and other international experts. Each trial that recruited over 1000 women will be invited to have a representative on the Advisory Group. This group will not have regular meetings, but may be consulted from time to time by means of email or teleconference, and may have occasional *ad hoc *meetings. Co-chairs of the Advisory Group: C Redman, C Roberts.

#### c. Collaborative Group

All potentially eligible trialists will be contacted and invited to become members of the Collaborative Group. The corresponding author for each study will be contacted in the first instance. If there is no response, the associated statistician, data manager and/or other authors will be contacted. This process will be updated annually for the duration of the project, to ensure that new trialists are offered the opportunity to join the project and contribute their data.

#### d. Project coordination centre

The project will be coordinated from the Centre for Perinatal Health Services Research (CPHSR), University of Sydney, NSW, Australia. The coordination centre will be responsible for the daily management of the project including correspondence, newsletter production, maintaining current trialist contact information and meeting/teleconference organisation.

#### e. Data management centre

The data management centre, based at the UK Cochrane Centre, will be responsible for the receipt, storage, and analysis of project data as directed by the Collaborative Group via the Steering Group.

#### f. Collaborators' meetings

All members of the Collaboration, including the Steering Group, the Advisory Group, and representatives of each participating trial, will be invited to attend regular Collaborators' meetings. These meetings will be scheduled, where possible, to coincide with the biennial International Society for the Study of Hypertension Pregnancy (ISSHP) congresses. The meetings will be designed to allow maximum input from the participating trialists into the design, conduct, analysis and reporting of the project's results. The final Collaborators' meeting, at which the results will be presented for discussion, is scheduled for 2006 in Oxford, UK. The discussion at this meeting will provide the basis for the paper publication.

### Funding

The National Health and Medical Research Council (NHMRC) of Australia have provided funding for the project through the Centre for Perinatal Health Services Research, University of Sydney. These funds are: a three year project grant (ID: 253636) for the overall project administration base in Sydney, and a Sidney Sax Public Health Postdoctoral Fellowship (ID: 245521), based in Oxford and Sydney. Additional support is being provided by the Resource Centre for Randomised Trials and the UK Cochrane Centre, located in Oxford, UK, and the Medical Research Council Clinical Trials Unit in London, UK.

### Publication policy

The results of the project's analyses will be presented to, and discussed with, the Collaborative Group before publication. The aim of publication will be presentation of the results, rather than their interpretation. The main manuscript will be prepared by the Secretariat, and then circulated to the Steering and Advisory Groups for comment and revision. The revised draft paper then will be circulated to all members of the Collaborative Group for comment before publication. All publications using these data will be authored in the name of the PARIS Collaboration, as follows: Perinatal Antiplatelet Review of International Studies (PARIS) Collaboration.

## Discussion

Despite good evidence that antiplatelet agents (principally low dose aspirin) reduce the incidence of pre-eclampsia and its consequences, such as preterm birth and perinatal mortality, uncertainty remains regarding whether some women (in terms of risk) benefit more than others, when in pregnancy treatment should ideally start, and whether treatment effectiveness is dependent on antiplatelet dose.

The best way to answer these questions is to utilise existing individual patient data from all women enrolled in trials that have addressed this question. This approach has been described as the 'gold standard' of systematic review methodology as it allows for more powerful and flexible analysis of both subgroups and outcomes.

The PARIS Collaboration has been formed to undertake a systematic review of all available trials, with meta-analysis based on individual patient data, to answer these important clinical questions. This will be the first individual patient data review in the perinatal field. Provision of data by the participating Collaborators commenced in 2004, and results will be ready for presentation in 2006. Following consultation and discussion with the Collaborative Group, the main publication is expected in early 2007.

## Competing interests

The author(s) declare that they have no competing interests.

## Authors' contributions

All authors, the named members of the PARIS Collaboration Steering Group, contributed to the development of the protocol, and read and approved the final manuscript.

## Pre-publication history

The pre-publication history for this paper can be accessed here:



## Supplementary Material

Additional File 1 List of eligible trials text document listing potentially eligible trials Click here for file

Additional File 2 Suggested coding sheet table listing variables and suggested coding Click here for file

Additional File 3 Data provision form form to collect trial level data and data provision procedures Click here for file
