# Supplementary material for: Antiplatelet agents for prevention of pre-eclampsia and its consequences: a systematic review and individual patient data meta-analysis
Source: BMC Pregnancy Childbirth. 2005 Mar 18;5:7. doi: 10.1186/1471-2393-5-7 (PMC555958; doi:10.1186/1471-2393-5-7)
Supplement: Additional File 1 — List of eligible trials text document listing potentially eligible trials [file 1471-2393-5-7-S1.pdf]

PARIS Collaboration: eligible trials as at January 2005

Trial identifier	Main citation
Australia 1988	Trudinger BJ, Cook CM, Thompson RS, Giles WB, Connelly AJ. Low-dose aspirin improves fetal weight in umbilical placental insufficiency. Lancet 1988;2:214-215.
Australia 1993	Michael CA, Walters BNJ. Low-dose aspirin in the prevention of pre-eclampsia: current evaluation. In: Eng-Soon T Shan Ratnam S, MacNaughton M (eds). Maternal physiology and pathology. The current status of gynaecology and obstetrics series. Vol 4. Carnforth: Parthenon Publishing Group Limited, 1993:183-189.
Australia 1995	Newnham JP, Godfrey M, Walters BJN, Philips J, Evans SF. Low dose aspirin for the trreatment of fetal growth restriction: a randomised controlled trial. Aust NZ J Obstet Gynaecol 1995;35:370-374.
Australia 1995a	Kincaid-Smith P, North RA, Fairley KF, Kloss M, Ihle B. Prevention of pre-eclampsia in high risk women with renal disease: a prospective randomised trial of heparin and dipyridamole. Nephrology 1995;1:297-300.
Australia 1996	Morris JM, Fay RA, Ellwood DA, Cook C, Devonald KJ. A randomized controlled trial of aspirin in patients with abnormal uterine artery blood flow. Obstet Gynecol 1996;87:74-78.
Australia 1996a	Ferrier C, North R, Kincaid-Smith P. Low dose aspirin delays the onset of pre-eclampsia in pregnancies with abnormal uteroplacental circulation. Proceedings of the 10th world congress of the International Society for the Study of Hypertension in Pregnancy, Seattle, Washington, USA. 4-8 August 1996;151.
Australia 1997	Gallery EDM, Ross MR, Hawkins M, Leslie GI, Gyory AZ. Low-dose aspirin in high-risk pregnancy. Hypert Preg 1997;16:229-238.
Austria 1992	Schrocksnadel H, Sitte B, Alge A, Stechel-Berger G, Schwegel P, Pastner E, Daxenbichler G, Hansen H, Dapunt O. Low-dose aspirin in primigravidae with positive roll-over test. Gynecol Obstet Invest 1992;34:146-150.
Barbados 1998	Rotchell YE, Cruickshank JK, Phillips Gay M, Griffiths J, Stuart A, Farrell B, Ayers S, Hennis A, Grant A, Duley L, Collins R. Barbados low dose aspirin study in pregnancy (BLASP): a randomized controlled trial for the prevention of pre-eclampsia an[illegible] its complications. Br J Obstet Gynaecol 1998;105:286-292.

Trial identifier	Main citation
Brazil 1996	ECPPA: randomised trial of low dose aspirin for the prevention of maternal and fetal complications in high risk pregnancies. Br J Obstet Gynaecol 1996;103:39-47.
China 1996	Wang Z, Li W. A prospective randomized placebo-controlled trial of low dose aspirin for prevention of intra uterine growth retardation. Chin Med J 1996;109:238-242.
China 1999	Rogers MS, Fung HYM, Hung CY. Calcium and low-dose aspirin prophylaxis in women at high risk of pregnancy-induced hypertension. Hyperten Preg 1999;18:165-172.
CLASP 1994	CLASP (Collaborative Low-dose Aspirin Study in Pregnancy) Collaborative Group. CLASP: a randomised trial of low-dose aspirin for the prevention and treatment of pre-eclampsia among 9364 pregnant women. Lancet 1994;343:619-629.
Colorado 1993	Porreco RP, Hickok DE, Williams MA, Krenning C. Low-dose aspirin and hypertension in pregnancy. Lancet 1993;341:312.
EPREDA 1991	Uzan S, Beaufils M, Breart G, Bazin B, Capitant C, Paris J. Prevention of fetal growth retardation with low-dose aspirin: findings of the EPREDA trial. Lancet 1991;337:1427-1431.
ERASME 2003	ERASME collaborative group. Aspirin (100mg) used for prevention of pre-eclampsia in nulliparous women: the Essai Regional Asprine Mere-Enfant study (Part 1). BJOG 2003, 110 (5):475-484.
Finland 1993	Viinikka L, Hartikainen-Sorri AL, Lumme R, Hiilesmaa V, Ylikorkala O. Low dose aspirin in hypertensive pregnant women: effect on pregnancy outcome and prostacyclin-thromboxane balance in mother and newborn. Br J Obstet Gynaecol 1993;100:809-815.
Finland 1997	Zimmerman P, Eirio V, Koskinen J, Niemi K, Nyman R, Kujansuu E, Ranta T. Effect of low dose aspirin treatment on vascular resistance in the uterine, uteroplacental, renal and umbilical arteries - a prospective longitudinal study on a high risk population with persistent notch in the uterine arteries. Eur J Ultrasound 1997;5:17-30.
Finland 2002	Vainio M, Kujansuu E, Iso-Mustajarvi M, Maenpaa J. Low dose acetylsalicylic acid in prevention of pregnancy-induced hypertension and intrauterine growth retardation in women with bilateral uterine artery notches. BJOG 2002; 109:161-167.

Trial identifier	Main citation
France 1985	Beaufils M, Uzan S, Donsimoni R, Colau JC. Prevention of pre-eclampsia by early antiplatelet therapy. Lancet 1985;1:840-842.
France 1990	Azar R, Turpin D. Effect of antiplatelet therapy in women at high risk for pregnancy-induced hypertension. Proceedings of 7th World Congress of Hypertension in Pregnancy, Perugia, Italy. 1990, 257.
Germany 2000	Grab D. Paulus WE, Erdmann M, Terinde R, Oberhoffer R, Lang D et al. Effects of low-dose aspirin on uterine and fetal blood flow during pregnancy: results of a randomized, placebo-controlled, double-blind trial. Ultrasound in Obstetrics and Gynecology 2000;15:19-27.
India 1993	Rai U, Chakravorty M, Juneja Y. Role of low dose aspirin in PIH. J Obstet Gynaecol India 1993;883-886.
India 1994	Roy UK, Pan S. A study of low dose aspirin in prevention of pregnancy induced hypertension. J Indian Med Assoc 1994;92:188-191.
India 1999	Shenoy S, Chandrika D, Pisharody R. RCT of low dose aspirin to prevent the progression of pregnancy induced hypertension Grade A to B. J Clin Epidemiol 1999;52 Suppl 1:28S.
Iran 2002	Taherian Ali-Akbar, Taherian A, Shirvani A. Prevention of preeclampsia with low-dose aspirin or calcium supplementation. Archives of Iranian Medicine, 2002; 5(3):151-156.
Israel 1989	Schiff E, Peleg E, Goldenberg M, Rosenthal T, Ruppin E, Tamarkin M, Barkai G, Ben-Baruch G, Yahal I, Blankstein J, Goldman B, Mashiach S. The use of aspirin to prevent pregnancy-induced hypertension and lower the ratio of thromboxane A2 to prostacyclin in relatively high risk pregnancies. N Engl J Med 1989;321:351-356.
Israel 1990	Schiff E, Barkai G, Ben-Baruch G, Mashiach S. Low-dose aspirin does not influence the clinical course of women with mild pregnancy-induced hypertension. Obstet Gynecol 1990;76:742-744.
Israel 1994	Caspi E, Raziel A, Sherman D, Arieli S, Bukovski I, Weintraub Z. Prevention of pregnancy induced hypertension in twins by early administration of low-dose aspirin: A preliminary report. Am J Reprod Immun 1994;31:19-24.

Trial identifier	Main citation
Italy 1989	Benigni A, Gregorini G, Frusca T, Chiabrando C, Ballerini S, Valcamonico A, Orisio S, Piccinelli A, Pinciroli V, Fanelli R, Gastaldi A, Remuzzi G. Effect of low-dose aspirin on fetal and maternal generation of thromboxane by platelets in women at risk for pregnancy- induced hypertension. N Engl J Med 1989;321:357-362.
Italy 1993	Italian Study of Aspirin in Pregnancy. Low-dose aspirin in prevention and treatment of intrauterine growth retardation and pregnancy-induced hypertension. Lancet 1993;341:396-400.
Italy 1999	Volpicelli T, D'Anto V, Faticato A, Galante L, Civitillo RM, Rappa C et al. Trial prospettico sull'uso profilattico dell'aspirina in donne gravide ad alto rischio di preeclampsia. Gestosi ’99 1999;159-160.
Italy 2004	Chiaffarino F, Parazzini F, Paladini D, Acaia B, Ossola W, Marozio L, Facchinetti F, Del Guidice A. A small randomised trial of low dose aspirin in women at high risk of pre-eclampsia. Eur J Obs Gynae and Reproductive Biology 2004, 112(2):142-144.
Jamaica 1998	Golding J. A randomised trial of low dose aspirin for primiparae in pregnancy. Br J Obstet Gynaecol 1998;105:293-299.
Japan 1999	Seki H, Kuromaki K, Takeda S, Kinoshita K, Satoh K. Trial of prophylactic administration of TXA2 synthetas inhibitor, ozagrel hydrochloride, for preeclampsia. Hyper Preg 1999; 18:157-164.
Netherlands 1986	Wallenburg HCS, Dekker GA, Makovitz JW, Rotmans P. Low-dose aspirin prevents pregnancy-induced hypertension and pre-eclampsia in angiotensin-sensitive primigravidae. Lancet 1986;1:1-3.
Netherlands 1989	Dekker GA. Prediction and prevention of pregnancy-induced hypertensive disorders: a clinical and pathophysiologic study. MD thesis, University Medical School Rotterdam, The Netherlands. 1989; 1-150.
Netherlands 1991	Wallenburg HCS, Dekker GA, Makovitz JW, Rotmans N. Effect of low-dose aspirin on vascular refractoriness in angiotensin-sensitive primigravid women. Am J Obstet Gynecol 1991;164:1169-1173.
New Zealand 1999	McCowan LME, Harding J, Roberts A, Barker S, Ford C, Stewart A. Administration of low dose aspirin to mothers with small for gestational age fetuses and abnormal umbilical Doppler studies to increase birthweight: a randomised double-blind controlled trial. Br J Obstet Gynaecol 1999;106:647-651.

Trial identifier	Main citation
PERGAR 1987	Uzan S, Beaufils M, Bazin B, Danays T. Idiopathic recurrent fetal growth retardation and aspirin-dipyridamole therapy [letter]. Am J Obstet Gynaecol 1989;160:763-4.
South Africa 1988	Railton A, Davey A. Aspirin and dypyridamole in the prevention of pre-eclampsia: effect on plasma prostanoids 6 keto PG1a and TXB2 and clinical outcome of pregnancy. Proceedings of the 6th world congress of the International Society for the Study of Hypertension in Pregnancy, Toronto, Canada. 1988;60.
Spain 1997	Hermida RC, Ayala DE, Iglesias M, Mojon A, Silva I, Ucieda R, Fernandez JR. Time-dependant effects of low dose aspirin administration on blood pressure in pregnant women. Hypertension 1997;30:589-595.
Spain 2003	Hermida RC, Ayala DE, Iglesias M. Administration time-dependent influence of aspirin on blood pressure in pregnant women. Hypertension 2003;41(Pt2):651-656.
Tanzania 1995	Ramaiya C, Mgaya HN. Low dose aspirin in prevention of pregnancy-induced hypertension in primigravidae at the Muhimbili Medical Centre, Dar es Salaam. East Afr Med J 1995;72:690-693.
Thailand 1996	Herabutya Y, Jetsawangsri T, Saropala N. The use of low-dose aspirin to prevent preeclampsia. Int J Gynecol Obstet 1996;54:177-178.
UK 1990	McParland PJ, Pearce JM, Chamberlain GVP. Doppler ultrasound and aspirin in recognition and prevention of pregnancy-induced hypertension. Lancet 1990;335:1552-55.
UK 1992	Louden KA, Broughton Pipkin F, Symonds EM, Tuohy P, O'Callaghan C, Heptinstall S, Fox S, Mitchell JRA. A randomized placebo-controlled study of the effect of low dose aspirin on platelet reactivity and serum thromboxane B2 production in non-pregnant women, in normal pregnancy, and in gestational hypertension. Br J Obstet Gynaecol 1992;99:371-376.
UK 1992b	Quenby S, Farquharson R, Ramsden G. The obstetric outcome of patients with positive anticardiolipin antibodies: aspirin vs no treatment. Proceedings of 26th British Congress of Obstetrics and Gynaecology, Manchester, U.K. 1992;443.

Trial identifier	Main citation
UK 1995	Davies NJ, Gazvani MR, Farquharson RG, Walkinshaw SA. Low-dose aspirin in the prevention of hypertensive disorders of pregnancy in relatively low-risk nulliparous women. Hyper Preg 1995;14:49-55.
UK 2003	Yu C.K.H, Papageorghiou AT, Parra M, Palma D, Nicolaides KH. Randomised controlled trial using low dose aspirin in the prevention of preeclampsia in women with abnormal uterine artery doppler at 23 weeks gestation. Ultrasound Obst Gynae 2003;22:233-239.
USA 1993	Hauth JC, Goldenberg RL, Parker CR Jr, Philips JB 3rd, Copper RL, DuBard MB, Cutter GR. Low-dose aspirin therapy to prevent preeclampsia. Am J Obstet Gynecol 1993;168:1083-1091.
USA 1993a	Sibai BM, Caritis SN, Thom E, Klebanoff M, McNellis D, Rocco L, Paul RH, Romero R, Witter F, Rosen M, Depp R, National Institute of Child Health and Human Development Network of Maternal-Fetal Medicine Units. Prevention of preeclampsia with low-dose aspirin in healthy, nulliparous pregnant women. N Engl J Med 1993;329:1213-1218.
USA 1994	August P, Helseth G, Edersheim TG, Hutson JM, Druzin M. Sustained release, low-dose aspirin ameliorates but does not prevent preeclampsia (PE) in a high risk population. Proceedings of 9th International Congress, International Society for the Study of Hypertension in Pregnancy, Sydney, Australia. 1994;72.
USA 1998	Cartis S, Sibai B, Hauth J, Lindheimer M, Klebanoff M, Thom E, VanDorten P, Landon M, Paul R, Midovnik M, Meis P, Thunau G and the National Institute of Child Health and Human Development Network of Maternal-Fetal Medicine Units. Low - dose aspirin to prevent preeclampsia in women at high risk. N Eng J Med 1998;338:701-705.
Venezuela 2000	Rivas-Echeverria CA, Echeverria Y, Molina L, Novoa D. Synergic use of aspirin, fish oil and vitamins C and E for the prevention of preeclampsia. Hypertension in Pregnancy 2000;19:30.
Zimbabwe 1998	Byaruhanga RN, Chipato T, Rusakaniko S. A randomized controlled trial of low-dose aspirin in women at risk from pre-eclampsia. Int J Gynaecol Obstet 1998;60: 129-135.
