# Supplementary material for: Antiplatelet agents for prevention of pre-eclampsia and its consequences: a systematic review and individual patient data meta-analysis
Source: BMC Pregnancy Childbirth. 2005 Mar 18;5:7. doi: 10.1186/1471-2393-5-7 (PMC555958; doi:10.1186/1471-2393-5-7)
Supplement: Additional File 2 — Suggested coding sheet table listing variables and suggested coding [file 1471-2393-5-7-S2.pdf]

PARIS Perinatal Antiplatelet Review of International Studies Collaboration

If possible, please use the suggested coding below when submitting your trial data

Enrolment characteristics

Variable name	Suggested coding
patient ID	unique patient ID used within your trial (preferably anonymous, not a name)
date randomisation*	date of randomisation
EDD	estimated date of delivery
LMP	date of first day of last menstrual period Please supply if EDD unknown.
gestation at randomisation	in completed weeks; or 9=unknown Please supply if EDD or LMP unknown.
allocated treatment	1=active treatment; 2=control
maternal date of birth	date of birth of enrolled woman
maternal age	maternal age in whole years, if maternal date of birth unknown
any previous pregnancy	0=no; 1=yes; 9=unknown
systolic blood pressure	systolic BP at randomisation, in mmHg, or 9=unknown
diastolic blood pressure	diastolic BP at randomisation, in mmHg, or 9=unknown
diagnosis raised BP	0=no; 1=yes; 9=unknown
proteinuria	0=no; 1=yes; 9=unknown
oedema	0=no; 1=yes; 9=unknown
risk factors	
multiple pregnancy	0=no; 1=yes; 9=unknown
autoimmune disease	0=no; 1=yes; 9=unknown
renal disease	0=no; 1=yes; 9=unknown
chronic hypertension	0=no; 1=yes; 9=unknown
diabetes	0=no; 1=yes; 9=unknown
previous PIH #	0=no; 1=yes; 9=unknown
previous PE^ / eclampsia	0=no; 1=yes; 9=unknown
previous early onset PE	0=no; 1=yes; 9=unknown
family history of PE	0=no; 1=yes; 9=unknown
abnormal Doppler flow❖	0=no; 1=yes; 9=unknown
previous SGA / IUGR✠	0=no; 1=yes; 9=unknown
previous fetal or neonatal loss	0=no; 1=yes; 9=unknown
unspecified risk	0=no; 1=yes; 9=not applicable Code as 'yes' if any of the above risk factors=yes, or if your trial only collected 'any risk factor', not specific risk factors.

Maternal outcomes

Variable name	Suggested coding
hypertension during this pregnancy	0=no; 1=yes; 9=unknown
highest recorded systolic BP	excluding during labour, in mmHg
highest recorded diastolic BP	excluding during labour, in mmHg
severe hypertension during this pregnancy	0=no; 1=yes; 9=unknown BP ≥160/110 or as defined in your trial
proteinuria during this pregnancy	0=no; 1=yes; 9=unknown
date of onset of proteinuria	date when proteinuria was first recorded
gestation at onset of proteinuria	If date of onset of proteinuria unavailable, please specify gestation at onset.
oedema this pregnancy	0=no; 1=yes; 9=unknown
pre-eclampsia	0=no; 1=yes; 9=unknown
drugs for pre-eclampsia during this pregnancy	0=no; 1=antihypertensives; 2=anticonvulsants; 9=unknown
severe maternal morbidity	1=none; 2=CVA/stroke; 3=renal failure; 4=liver failure; 5=pulmonary oedema; 6=disseminated intravascular coagulation; 7=HELLP syndrome; 8=eclampsia; 9=not recorded
onset of labour	1=spontaneous; 2=induced; 3=pre-labour Caesarean Section; 9=not recorded
delivery mode	1=spontaneous vaginal; 2=assisted vaginal; 3=vaginal unspecified; 4=Caesarean Section; 9=not recorded
antepartum haemorrhage	0=none; 1=yes, but type not specified; 2=placental abruption only; 3=other; 9=not recorded
estimated blood loss at delivery	in ml if unknown, please record PPH below:
postpartum haemorrhage	0=no; 1=yes; 9=not recorded
maternal death	0=no; 1=yes; 9=not recorded
excluded from main analyses	0=no; 1=yes; 9=unknown Was this woman excluded from your main analyses?
reason for exclusion	If the woman was excluded from the main analyses of your trial, please state reason.

Please turn over for further variables and notes: →

For further information, contact: **Lisa Askie** **laskie@cochrane.co.uk** **Phone: +44 (0)1865 516 300** **Direct: +44 (0)1865 517 621**

If possible, please use the suggested coding below when submitting your trial data

Fetal / neonatal / child outcomes

Variable name	Suggested coding	Variable name	Suggested coding
DOB	date of birth	admitted to SCN or NICU	0=no; 1=yes; 9=unknown. Was this infant admitted to a Special Care Nursery (SCN) or Neonatal Intensive Care Unit (NICU)?
GA	gestation at birth, in completed weeks	assisted ventilation	0=no; 1=yes; 9=unknown. Did this infant receive positive pressure ventilation of any type, for any duration?
BW	birthweight, in grams	date of hospital discharge	date of discharge from first hospitalisation episode, if known
sex	1=male; 2=female; 3=ambiguous; 9=unknown	days in hospital	If date of discharge unknown, please supply number of days in hospital during initial hospitalisation episode, if known.
SGA / IUGR ✠	0=no; 1=yes; 9=unknown<br><br>Please supply details of definition and growth charts used.	neonatal bleeding	1=none; 2=intra/periventricular; 3=cord; 4=gastro-intestinal; 5=pulmonary; 6=cephalohaematoma / bruising; 7=other site; 8=yes, but site unspecified; 9=unknown or not recorded
alive at birth	1=alive at birth and survived; 2=not alive at birth; 3=alive at birth and died; 9=not recorded	neonatal other bleeding type	If 'other site', please describe site of bleeding.
date of death	If fetal/infant death occurred, please supply date of fetal/infant death, if known.	any post discharge followup information on this infant	Please supply any other information recorded regarding post-discharge follow-up of the infant.
age at death	If date of death unknown, please supply age at fetal/infant death, if known.	any other fetuses/infants from this pregnancy	0=no; 1=yes; 9=unknown

If there was more than one fetus / infant from this pregnancy, please supply the same data as above for each fetus / infant.
Please use the same variables as above, but name the fields: 2DOB, 2GA and so on.

Notes:

- * Dates should be in the format: dd/mm/yyyy or dd/mm/yy
- # PIH: pregnancy induced hypertension, also known as gestational hypertension
- ^ PE: pre-eclampsia
- ❖ abnormal Doppler flow refers to abnormal uterine artery Doppler flow studies
- ✠ SGA: small for gestational age at birth, also known as IUGR: intrauterine growth retardation/restriction Please supply your definition of SGA/IUGR and information regarding any local charts or norms used to calculate this outcome. If possible, please supply copies of any such charts.
- Please refer to the enclosed protocol for more detailed definitions if required.
