# Supplementary material for: Antiplatelet agents for prevention of pre-eclampsia and its consequences: a systematic review and individual patient data meta-analysis
Source: BMC Pregnancy Childbirth. 2005 Mar 18;5:7. doi: 10.1186/1471-2393-5-7 (PMC555958; doi:10.1186/1471-2393-5-7)
Supplement: Additional File 3 — Data provision form form to collect trial level data and data provision procedures [file 1471-2393-5-7-S3.pdf]

PARIS individual patient data meta-analysis: data provision form

Trial ID: __________________ **Name of person completing this form:** _________________________

Your email: _______________________________________ **Your fax number:** _____________________

General information about your trial

Was informed consent obtained from each woman? Yes ☐ No ☐

Date trial opened to accrual: ____ / ____ / ____ (d d / mm / y y) Date trial closed to accrual: ____ / ____ / ____ (d d / mm / y y)

At the time the trial closed to accrual, what were the total number of women randomised? ______________

Have data for all women randomised into the trial been published / reported? Yes ☐ No ☐

Please list the **treatments used in each arm of your trial** (include drug dosages, intended gestation at cessation of treatment and/or length of treatment, and any other relevant standard local treatments):

Active treatment arm:____

____

Control arm: ____

____

Definitions used in your trial

Please supply information regarding how you defined the following outcomes in your trial. If possible, provide a *copy of the full trial protocol* as well.

Proteinuria: ____

Raised blood pressure / hypertension: ____

Pre-eclampsia: ____

Small for gestation age / intrauterine growth restriction or retardation (including which normative charts used):

____

Duration of intended treatment for the trial intervention: ____

Information about the design of your trial

What method was used to generate the random allocations in the trial?

Simple (such as coin toss) ☐ Random number tables ☐ Permuted blocks ☐ Minimisation ☐

Other ☐ If other, please state method used: ____

What, if any, stratification factors were used? ____

What proportions was the trial designed to have in each arm? (for example, 1:1) ____________________

What method was used to conceal the random allocation?

Opaque, sequentially numbered, sealed envelopes ☐ Central telephone ☐

Other ☐ If other, please state method used: ____

Please turn over for further information: →

Please return, by 1st October 2004, to: **Dr Lisa Askie, PARIS Collaboration, UK Cochrane Centre, Summertown Pavilion, Middle Way, Oxford, OX2 7LG, UK**

Email: laskie@cochrane.co.uk **Fax: +44 (0)1865 516 311** **Phone: +44 (0)1865 516 300**
Ftp site: ftp://paris@ftp.cochrane.co.uk **Direct: +44 (0)1865 517 621**

PARIS individual patient data meta-analysis: data provision form

Data transfer information

Please provide the data listed on the attached sheet, on all women randomised, if you collected these variables in your trial. This includes any women randomised whose data did not appear in published reports, regardless of whether or not they were included in the analyses.

Data can be supplied in almost any format (Excel, Access, Dbase, FoxPro, ASCII etc), but please indicate below which format has been used. It would be helpful if you used the coding suggested in the attached document, however, you may code the data in the way that is most convenient to you. Please supply us with full details of the data coding system used. If you are able to supply the full trial protocol and/or sample blank data collection sheets, this would also be very helpful.

What format have you supplied data in? ____

Have you used the suggested coding for the data you are supplying? Yes ☐ No ☐

If no, have you sent us details of the coding system you have used? Yes ☐ No ☐

Have you sent us a full trial protocol and/or blank data collection sheets? Yes ☐ No ☐

Our **preferred method for the transfer of data** to us is by lodging it on a secure ftp website at the data coordination centre (see below for instructions). However, please send us your data by any of the following methods if this is more convenient: completing new data forms (provided on request); sending either the original data forms, a computer printout of the data, or the data files on floppy disk (formatted for PC) or CD; or by emailing the data files to us. If you email the data file(s), please encrypt the data if possible and send the encryption key to us in a separate email.

What method of data transfer have you used?

Lodged data file(s)on ftp website ☐ Emailed data file(s) ☐ Sent data file(s) on disk via post ☐

Sent computer printout of data ☐ Sent original data forms ☐ Other: ______________________

What date did you send / lodge your data? ____ / ____ / ____
d d mm y y

To lodge your data on the secure PARIS ftp website:

Start Internet Explorer (or your web browser). Go to the website: ftp://paris@ftp.cochrane.co.uk

When prompted, enter the password **XparisX** You will then see other files that have already been lodged (but you won't be able to access them). Reduce the size of the browser window that is displaying the PARIS ftp website and open Windows Explorer. Size both windows so that you can see the contents of each.

Please name your file so it can be recognised as belonging to your trial, for example: Australia 2004.xls. To deposit your file, go to the appropriate folder in Windows Explorer, select it, and holding the left mouse key down (or right mouse for left hand users), drag and drop the file onto the ftp site. This will copy your file to the secure website. Close down the web browser and Windows Explorer.

Guarantee of confidentiality of individual trial results

Any data supplied will remain the property of the trialist(s) who supplied it. These data will remain confidential and will not be used, circulated or distributed in any way that allows access to individual trial data.

I wish my data to remain confidential Yes ☐ No ☐

Signed ____ Date ____________________________

Please return, by 1st October 2004, to: **Dr Lisa Askie, PARIS Collaboration, UK Cochrane Centre, Summertown Pavilion, Middle Way, Oxford, OX2 7LG, UK**

Email: laskie@cochrane.co.uk **Fax: +44 (0)1865 516 311** **Phone: +44 (0)1865 516 300**
Ftp site: ftp://paris@ftp.cochrane.co.uk **Direct: +44 (0)1865 517 621**
